# The Effects of Providing a Connected Scale in an App-Based Digital Health Program: Cross-sectional Examination

**DOI:** 10.2196/40865

**Published:** 2023-02-03

**Authors:** Lisa A Auster-Gussman, Mohit Rikhy, Kimberly G Lockwood, OraLee H Branch, Sarah A Graham

**Affiliations:** 1 Lark Health Mountain View, CA United States

**Keywords:** engagement, retention, scales, self-monitoring, mobile app, digital health, AI, smartphone, platform, app, application, health program, program

## Introduction

Self-monitoring technologies (eg, digital scales) have been shown to improve health outcomes [1,2], such as weight loss, when combined with additional interventions such as coaching [3]. This may be because they facilitate increased self-weighing, which has been shown to be related to better health outcomes [4,5]. The purpose of this study was to examine whether the provision of a digital body weight scale as part of one’s digital health program was related to increased self-weighing and longer retention. The primary hypothesis was that members provided with a scale would weigh more frequently and remain in the program for longer than those not provided with a scale.

## Methods

### Study Design

We conducted an observational study of members enrolled in an artificial intelligence (AI)–powered digital health program available via smartphone on a platform called Lark. Information about Lark is published elsewhere [5,6]. We examined differences in self-weighing and retention between members with and without scales provided by their commercial insurance provider. Members received their scales immediately after the completion of enrollment.

### Ethical Considerations

The study received exemption status from Advarra Institutional Review Board (protocol #Pro00047181) for retrospective analyses of previously collected and deidentified data.

### Participants, Program Description, and Inclusion Criteria

We conducted an analysis of 3488 members enrolled from 2019 to 2021 in a yearlong digital program focused on general health and well-being; weight loss was not a specific target. The program included automated personalized coaching via in-app messaging using conversational AI, weekly lessons related to healthy lifestyle choices, meal logging, and weekly weight logging. The inclusion criteria were age ≥18 years, ≥1 full year has passed since enrollment date, completion of ≥1 educational lesson in the first 6 months, and ≥1 weigh-in during the first 6 months.

### Outcome Measures

We examined two key outcome variables. Weigh-ins included the total number of days with recorded weigh-ins during each member’s first 6 months in the program. This included both weigh-ins from the provided scales that sync with the app automatically and manually entered weights. Scales not provided by Lark do not pair directly with the app, so weigh-ins on non-Lark scales would need to be entered manually in the app. We analyzed the first 6 months because this is the active weight loss phase of the program for members who set a goal to lose weight. The second 6 months is the maintenance phase. Active retention was the total number of days from the day the individual enrolled to the last day that they used in-app functions, such as conversation or meal logging, up to 365 days.

### Analysis

We calculated descriptive statistics for the overall sample and examined differences in weigh-ins and active retention between members provided with versus not provided with scales using analysis of covariance (ANCOVA).

## Results

### Participants

Participants were 68.3% (2384/3488) female with a mean age of 45.19 (SD 11.45) years. Of the 3314 members who reported a starting weight, the mean starting BMI was 31.1 (SD 6.93) kg/m^2^, and 48.6% (1611/3314) had obesity. Race/ethnicity data were not available for more than half of the sample and therefore not reported. Approximately 43.5% (1519/3488) of members received the insurance-provided scale, and the pairing rate was 93.7% (1423/1519).

### Associations Between Provision of a Digital Scale and Descriptive Statistics

Although the groups were similar in mean starting BMI (no scale mean 31.52, SD 7.25 kg/m^2^; scale mean 30.55, SD 6.44 kg/m^2^) and age (no scale mean 44.63, SD 11.35 years; scale mean 45.91, SD 11.55 years), the differences were statistically significant (starting BMI *t*_3312_=4.05, *P*<.001; age *t*_3816_=–3.22; *P*=.001). There was also a greater proportion of women among those not provided with a scale (1411/1969, 71.7%) than among those provided with a scale (973/1519, 64.1%; χ^2^_1_=22.93; *P*<.001). Therefore, we controlled for starting BMI, age, and sex in the ANCOVAs.

### Associations Between Provision of a Digital Scale, Weigh-ins, and Active Retention

The ANCOVAs revealed that members provided with versus not provided with a scale had significantly more days with weigh-ins and days of active retention (see Table 1); their mean last day with a weigh-in also occurred further into the program (no scale mean 72, SE 2 days; scale mean 138, SE 3 days). On average, members not provided with a scale weighed themselves 1-2 days per month and were retained for approximately 4 months, whereas members provided with a scale weighed themselves 1 day per week and were retained for almost 6 months.

**Table 1:** Analysis of covariance table of mean differences based on the provision of digital scale^a^.

Engagement features	No scale, mean (SE)	Scale, mean (SE)	*F* test (*df*)	*P* value
Days with weigh-ins during first 6 months	7.35 (0.24)	21.56 (0.66)	276.80 (1, 3202)	<.001
Days of active retention in first 12 months	120.44 (2.28)	172.45 (2.77)	15.74 (1, 3202)	<.001

^a^Analyses of covariance include age, sex, BMI, and days of active retention as control variables; 3208 members included in the analysis who had no missing data needed for analyses; 1822 members with no scale and 1386 members with scale.

## Discussion

These findings demonstrate that members provided with a scale recorded 3 times more days with weigh-ins and were retained almost 2 months longer than those not provided with a scale. The direct digital transfer of weights from the scale to the app greatly streamlined the weigh-in process, improving the member experience, which may have led to the observed longer retention. This research was limited by the fact that the provision of the scale was not individually randomized but the result of insurance providers’ choice to provide or not provide scales. In addition, although findings from past research show that both self-weighing and retention are associated with weight loss [4,5,7], suggesting that the provision of a scale might also be related to weight loss, this question was outside the scope of this analysis. This preliminary study is timely, given the increasing importance of understanding and attenuating the high rates of attrition in digital health [8-10], and might assist insurance providers to weigh the cost of provisioning a scale to the benefits of increased retention in lifestyle behavioral programs.

## Abbreviations

AI: artificial intelligence
ANCOVA: analysis of covariance
